# Liver X Receptors: Regulators of Cholesterol Metabolism, Inflammation, Autoimmunity, and Cancer

**DOI:** 10.3389/fimmu.2020.584303

**Published:** 2020-11-03

**Authors:** Maria Teresa Bilotta, Sara Petillo, Angela Santoni, Marco Cippitelli

**Affiliations:** ^1^ Department of Molecular Medicine, Sapienza University of Rome, Rome, Italy; ^2^ Istituto Pasteur-Fondazione Cenci Bolognetti, Rome, Italy; ^3^ Istituto Mediterraneo di Neuroscienze Neuromed, Pozzilli, Italy

**Keywords:** liver-X-receptor, cholesterol, inflammation, autoimmunity, cancer metabolism, antitumor immune responses

## Abstract

The interplay between cellular stress and immune response can be variable and sometimes contradictory. The mechanisms by which stress-activated pathways regulate the inflammatory response to a pathogen, in autoimmunity or during cancer progression remain unclear in many aspects, despite our recent knowledge of the signalling and transcriptional pathways involved in these diseases. In this context, over the last decade many studies demonstrated that cholesterol metabolism is an important checkpoint for immune homeostasis and cancer progression. Indeed, cholesterol is actively metabolized and can regulate, through its mobilization and/or production of active derivatives, many aspects of immunity and inflammation. Moreover, accumulation of cholesterol has been described in cancer cells, indicating metabolic addiction. The nuclear receptors liver-X-receptors (LXRs) are important regulators of intracellular cholesterol and lipids homeostasis. They have also key regulatory roles in immune response, as they can regulate inflammation, innate and adaptive immunity. Moreover, activation of LXRs has been reported to affect the proliferation and survival of different cancer cell types that show altered metabolic pathways and accumulation of cholesterol. In this minireview we will give an overview of the recent understandings about the mechanisms through which LXRs regulate inflammation, autoimmunity, and cancer, and the therapeutic potential for future treatment of these diseases through modulation of cholesterol metabolism.

## Introduction

Cholesterol metabolism is deeply linked to different aspects of immunity and inflammation. It is generally thought as an exogenous player on immunity during disease, as in the case of pathologic cholesterol overloading of foam cells in atherosclerosis or more in general in hypercholesterolaemia. However, increasing evidences have recently changed this view by demonstrating that a number of immune receptors and transcription factors such as Toll-like Receptors (TLRs), C-X-C motif chemokine receptor 2 (CXCR2), Stimulator of IFN genes (STING) and retinoic acid-related orphan receptor-γt (ROR-γt) are profoundly regulated by sterols (1–7). Moreover, regulation of intracellular cholesterol homeostasis controls lymphocyte proliferation and adaptive immune responses (8).

**Figure 1:**
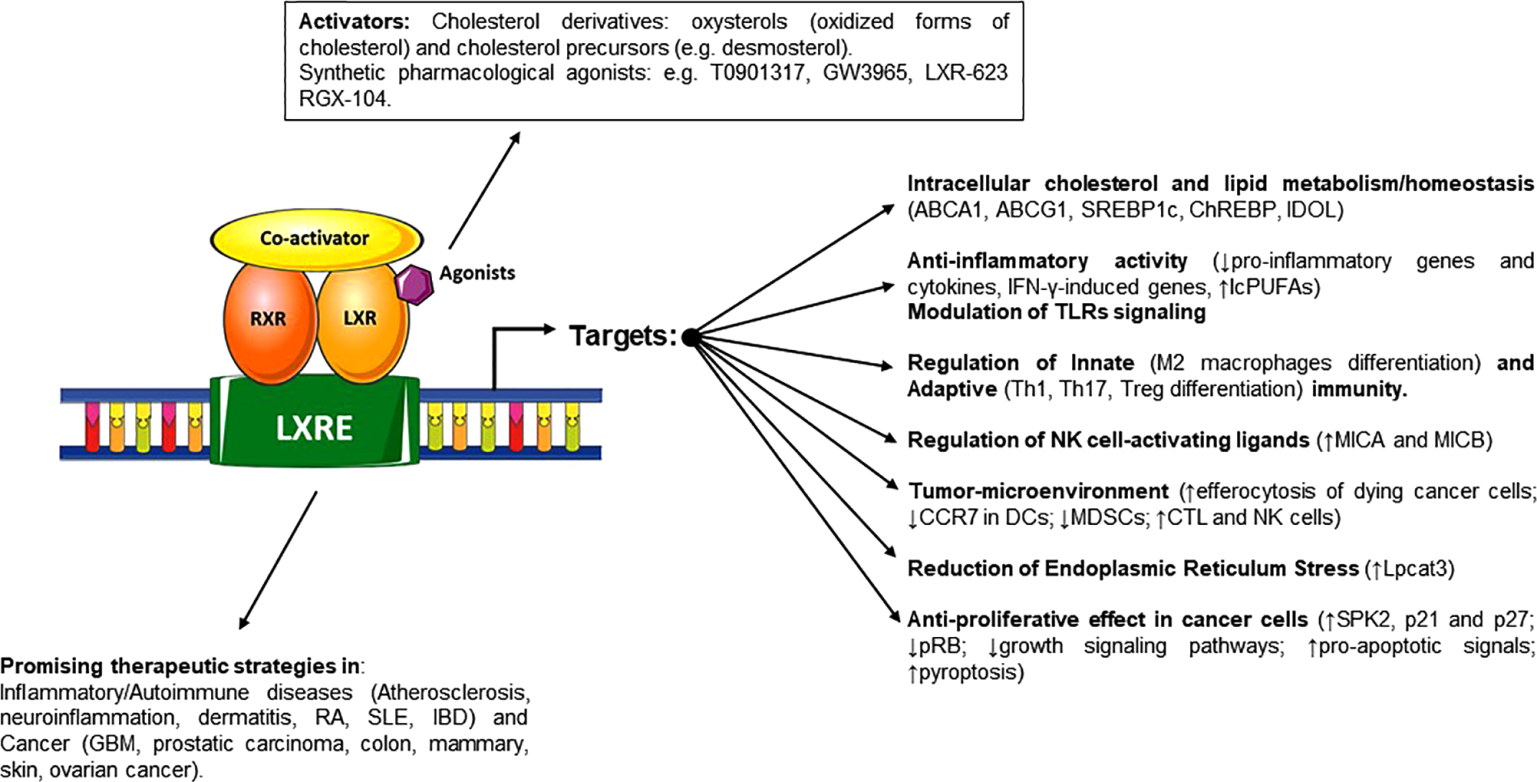
Schematic representation of LXRs activators and the different effects on regulated genes and pathways involved in cholesterol and lipid homeostasis, regulation of immune system and cancer proliferation and progression. When these ligands trigger the activation of LXRs, they heterodimerize with RXR and bind to target gene promoters on LXR-responsive-elements, regulating the transcription and expression of specific target genes.

In this review we will discuss recent literature regarding aspects of lipid and cholesterol metabolism in tissues homeostasis, providing to the readers a synthetic overview of the main connections and regulatory interactions between cholesterol cellular metabolism and the activity of LXRs in the context of inflammation, autoimmunity and cancer. LXRs are transcription factors able to regulate specific gene networks implicated in cholesterol and lipid metabolism both in homeostatic and pathological conditions. Moreover, LXRs can mediate anti-inflammatory activities and modulate the immune response, promoting the expression of mediators which have a role in the control of inflammatory disorders and in the response to microbial infection. In a different scenario, accumulation of cholesterol has been also described in many types of cancer cells indicating metabolic addiction. This further expands the possible implications of its dysregulation in cancer progression (9, 10), configuring cholesterol as an important metabolic determinant. LXRs play relevant roles in cancer biology and in anti-tumor immune responses, opening new therapeutic possibilities (**Figure 1**) and (**Table 1**).

**Table 1:** Activities of LXR in inflammation, autoimmunity, and cancer.

LXR, Inflammation, and Autoimmunity		
LXR/cholesterol-mediated responses	Immune mechanisms	Experimental models
Cholesterol enrichment in macrophage plasma membrane promotes the activity of TLRs (11, 12).	Cholesterol crystals uptake in macrophages activate NLRP3/inflammasome, and the pro-inflammatory cytokines IL-1β and IL-18 (13).	Atherosclerosis susceptibility (14). Atherosclerosis plaque (13).
Upregulation of ABCA1 and ABCG1 on engulfed apoptotic cells (15, 16). Differentiation of M2 macrophages (15, 16).	Prevention immune system anomalous activation (15, 16).	Efferocytosis (15, 16).
Transrepression: LXR binds to the NCoR-SMRT co-repressor preventing signal-dependent clearance from the promoter of pro-inﬂammatory genes (17, 18).	Transcriptional repression of NF-kB, AP-1, STAT1. Inhibition of primary cytokine production (17, 19, 20). Repression of pro-inflammatory cytokine maturation to their active form (e.g., IL-18) (21).	Inflammation and autoimmune diseases (atherosclerosis, dermatitis, neuroinﬂammation, lupus and arthritis) (22–23).
Indirect activity on inflammation: induction of lcPUFAs (e.g., omega 3 fatty acid) (24).	Decrease of transactivation mediated by NF-kB of inflammatory genes (24).	Inflammation control.
LXRα maintains BBB integrity and its activation modulates the pro-inflammatory response in astrocytes/microglia (25–27). Activation of LXR leads SREBP-1 to act on IL-17 promoter (28).	Reduced production of the pro-inflammatory cytokines IL-17 and IFN-γ and reduced expression of IL-23R (28, 29).	Demyelinating disease (30).
Activation of LXR by pharmacologic agonists or ligands present in synovial fluid.	Decreased pro-inflammatory cytokines production in CIA models (23, 31–32). Enhanced TLR-driven cytokines and chemokines secretion in RA synovitis (33–34).	Rheumatoid Arthritis (35). CIA models (23, 31–32).
LXR activation mediates anti-inflammatory effects in colon epithelial cells (36). Lack of LXR induces colitis in DSS and TNBS murine models.	LXR activation can suppress Th1 and Th17 polarization *in vitro* and promote the differentiation of gut associated Tregs (37).	Intestinal bowel disease (36, 37).
**LXR and Cancer**		
**LXR-mediated cellular response**	**Immune mechanisms**	**Cancer models and LXR activity**
Induction of cholesterol efflux and reduction of its uptake with consequent reduced tumor cell proliferation and survival (38–39). Reduced expression/activity of cell-cycle regulators (SPK2) (40), higher expression of cell-cycle inhibitors (p21, p27) and decreased phospho-RB protein levels (41, 42). Delayed progression of androgen-dependent tumors towards androgen independence (41, 42).	Decrease MDSCs through the induction of ApoE and potentiate activation of cytotoxic lymphocytes (43). Oxysterols impairs DC migration through the inhibition of CCR7 (44). Activation of LXRα in macrophages stimulates phagocytosis of dying cancer cells (45). LXR upregulates the expression of the NKG2D ligands MICA and MICB in MM and improved NK cell cytotoxicity (46).	Glioblastoma multiforme (38–39) Non-small-cell lung carcinoma (NSCLC) (9) Prostatic carcinoma (9) Ovarian cancer (9) Colon cancer (9) Mammary and Skin cancer (9) Multiple Myeloma (46)

The implications of direct LXR-mediated actions and regulation of cholesterol metabolism in the control of inflammatory diseases and cancer progression. This table summarizes the different experimental models and the roles of LXR in these pathologic conditions.

## LXRs: A Link Between Lipid Metabolism and Immune Response

LXRs are transcription factors belonging to the nuclear receptors (NRs) superfamily. They are master regulators of cholesterol and lipid intracellular homeostasis (47). There are two isoforms of LXRs, LXRα (NR1H3), and LXRβ (NR1H2) (48, 49) that share extensive sequence homology [(77% identity in both the DNA binding domain (DBD) and ligand binding domain (LBD)]. Despite this similarity, they have rather different expression patterns (50); indeed, the expression of these NRs depends on the cell type and tissues analyzed, with LXRα more expressed in liver, intestine, adipose tissue and cells of the myelomonocytic lineage, while LXRβ is expressed more ubiquitously (51). Thus, their transcriptional role seems to be determined by their relative expression levels in specific tissues or cells, although important differences have also been identiﬁed in vivo between the two isoforms (52).

Different studies *in vitro* and *in vivo* have characterized a number of cholesterol derivatives including oxysterols, oxidized forms of cholesterol and cholesterol precursors (e.g., desmosterol) as LXR activators, able to bind with different affinities to the LXR LBD (47, 53, 54). When these endogenous ligands, or synthetic pharmacological agonists, trigger activation of LXRs, they heterodimerize with retinoid X receptors (RXR) and bind to target gene promoters on LXR-responsive-elements (LXREs), canonical binding sites composed of a repeated 6-mer sequence (5’-AGGTCA-3’) separated by four nucleotides (55). To activate target gene transcription, unliganded LXRs and co-repressors such as nuclear receptor corepressor 1 (NCoR1) and silencing mediator of retinoic acid and thyroid hormone receptor (SMRT), bound to LXREs, have to be displaced from chromatin to allow the binding of transcriptional co-activators [i.e., nuclear receptor co-activator 1 (NCOA1) and activating signal co-integrator 2 (ASC2)], leading to transcription (17).

Recent findings suggest that LXRs may be also recruited *de novo* to the promoter of target genes when triggered by ligands (18).

Once activated, they regulate the expression of genes involved in lipid and glucose metabolism (51, 56). In this context, LXRs are master regulators of cholesterol sensing; they counteract aberrant cellular sterol overload by upregulating the expression of sterol transporters such as the ATP binding cassette (ABC) family members ABCA1 and ABCG1, together with the transcription factors sterol regulatory element-binding protein 1c (SREBP1c) and carbohydrate-response element-binding protein (ChREBP) that regulate critical lipogenic pathways. Moreover, the activation of LXRs also induces the expression of inducible degrader of the LDL-receptor (IDOL), which is able to reduce the expression of low-density lipoprotein receptor (LDLR)s on the cell surface and the uptake of LDL/cholesterol particles (57).

Besides the regulation of cholesterol homeostasis, genetic and pharmacological studies have pointed out the role of LXRs as an important link between lipid metabolism, regulation of immune cell function and inflammation (58). Indeed, these NRs can both promote and repress the expression of specific immune regulatory gene networks (59). As discussed below, LXRs can induce anti-inflammatory activities in macrophages and Dendritic Cells (DCs) and represent a critical link between cholesterol metabolism, proliferation and migration of activated T and B lymphocytes (8, 15, 21, 28, 60–65), thus playing an important role in the control of inflammatory, autoimmune and infectious diseases.

## LXRs, Cholesterol, and Inflammation

Different pathways link inflammation to cholesterol metabolism and LXRs activity. Alteration of cellular cholesterol homeostasis can both enhance or reduce innate receptor signalling and inflammasome activation. Cholesterol enrichment in macrophage plasma membrane promotes the activity of TLRs as in the case of the TLR4-MD2 and TLR4-CD14 complexes activated in response to lipopolysaccharide (LPS) (11, 12). On the other hand, the activation of the reverse cholesterol transport (RCT) mediated by ABCA1 and ABCG1 transporters limits the formation of cholesterol-enriched lipid rafts in the plasma membrane and/or in the endosomal system. This inhibits MyD88-dependent TLRs trafficking by selective reduction of free cholesterol content and suppresses macrophage inflammatory responses (66). This mechanism has been elegantly demonstrated in mouse models deficient for ABCA1 and ABCG1, shown to accumulate cholesterol in peritoneal macrophages and to exhibit enhanced inflammatory responses to TLR agonists (11). In line with these observations, in a model of atherosclerosis susceptibility, pathogens can interfere with macrophage cholesterol metabolism through inhibition of the LXRs. Here, the activation of TLR-3 and -4 by microbial ligands has been shown to repress the expression of selected target genes including ABCA1 in macrophages, as clearly shown in aortic tissue *in vivo*, with a mechanism connected to reduced cholesterol efflux from macrophages regulated by interferon regulatory factor-3 (IRF3)-mediated inhibition of LXRs on their target promoters (14). Activation of efferocytosis is also associated to the activity of LXRs, which results in the efflux of free cholesterol derived from engulfed apoptotic cells by upregulating ABCA1 and ABCG1 transporters. This mechanism, together with the LXR-mediated alternative (M2) macrophage differentiation, can prevent aberrant activation of the immune system (15, 16). Moreover, the removal of apoptotic cells helps avoiding autoimmunity, as shown in murine models of lupus-like autoimmunity where treatment with LXR agonists ameliorated disease progression (15, 67). In a different context, increased cellular content of cholesterol can trigger cholesterol crystal formation, as shown in atherosclerotic plaques. In this disease model, cholesterol crystals uptake or formation in macrophages has been shown to activate NLR family pyrin domain containing 3 (NLRP3)/inflammasome with the secretion of the pro-inflammatory cytokines interleukin-1β (IL-1β) and IL-18 and to promote the progression of atherogenesis (13).

As shown for other NRs, LXRs are anti-inflammatory; they can inhibit the transcriptional induction of pro-inflammatory genes mediated by critical transcription factors as NF-kB, AP-1 or STAT-1. In this regard, pharmacological activation of LXRs has been shown to ameliorate the severity of the inﬂammatory response in murine models of atherosclerosis (22), neuroinﬂammation (30, 68), dermatitis (22, 69), lupus (67) and arthritis (23), inhibiting primary cytokine production. Mechanistically, studies using LXR agonists in macrophages have shown that, depending on the LXR isoform, these NRs can repress the induction of pro-inﬂammatory genes through a molecular mechanism known as “transrepression”. Here, after histone deacetylase-4 (HDAC-4)-dependent conjugation of LXR with small ubiquitin-related modifier (SUMO)-2/3 at speciﬁc lysine residues in the LBD, LXR becomes able to bind to the NCoR-SMRT co-repressor, thus preventing signal-dependent clearance from the promoters of pro-inﬂammatory genes (17, 19). With a different mechanism, LXRs can inhibit Interferon-γ-induced genes in astrocytes, where LXRα and LXRβ are SUMO-conjugated by HDAC4 or by protein inhibitor of activated STAT1 (PIAS1), respectively, and interact with phosphorylated signal transducer and activator of transcription-1 (STAT-1) preventing its binding to gene promoters (20). Furthermore, LXRs activation can repress pro-inflammatory cytokine maturation to their active form as demonstrated for IL-18 and can induce specific endogenous inhibitors (i.e., IL-18BP) (21).

In addition to direct transrepression activity on pro-inflammatory genes, LXRs can mediate other important integrated mechanisms contributing to the control of inflammation. LXRs can induce the synthesis of long-chain polyunsaturated fatty acids (lcPUFAs) such as omega 3 fatty acids. The presence of lcPUFAs can decrease transactivation mediated by NF-kB of inflammatory genes, modifying histone acetylation in their regulatory regions (24). Moreover, lcPUFAs have been shown to increase the production of eicosanoids and selected pro-resolving lipid mediators (70, 71). Interestingly, increased LXRs activity can also induce macrophage polarization toward a more pro-resolving phenotype, directly upregulating the expression of MER proto-oncogene Tyrosine Kinase (MERTK), a receptor that promotes the synthesis of mediators implicated in inflammation resolution (15, 72). Furthermore, as demonstrated in hepatic inflammation models, induction of the polyunsaturated phospholipids (PLs) remodeling enzyme lysophosphatidylcholine acyltransferase 3 (Lpcat3) by LXRs increases the formation of PLs and decreases membrane saturation, counteracting endoplasmic reticulum stress induced by fatty acids in hepatocytes, improving hepatic metabolic stress and inﬂammation by modulating aberrant c-Src activation (73). An additional consideration that can add a layer of complexity is that LXRs are highly expressed by haematopoietic stem cells (HSCs) and myeloid progenitor cells. In these cells, activation of LXRs can increase the ABCA1/ABCG1/apolipoprotein E (APOE)-mediated cholesterol efflux, which is able to reduce their proliferative responses to IL-3 and GM-CSF, thus indirectly modulating the production of inflammatory cells (74).

## LXRs and Autoimmunity

The activity of LXRs and cholesterol metabolites is implicated in the control and progression of several autoimmune diseases.

Altered lipid proﬁles have been associated with poor outcome of multiple sclerosis (MS) (75–80), an autoimmune disease characterized by inﬂammatory cell inﬁltrates and demyelination (81, 82). In this regard, obesity, among other environmental factors, has been described as a risk factor for MS in several epidemiological studies (83–86). In animal models of experimental autoimmune encephalomyelitis (EAE), the most common experimental model for human inflammatory demyelinating disease, selected agonists of LXRs (e.g., T0901317) have been shown to improve the severity of central nervous system inflammation (30). In line with this evidence, the activity of LXRα is indispensable for maintaining blood-brain barrier (BBB) integrity and its immune quiescence. Indeed, in a model of EAE, the speciﬁc knockout of LXRα in brain endothelial cells has been shown to increase BBB permeability and endothelial inﬂammation (25). Morover, activation of LXRs using agonists *in vivo* has been shown to repress the production of the pro-inflammatory cytokine IL-17 (28), together with IFNγ and IL-23R expression (29). Noteworthy, Th17 cell diﬀerentiation is modulated by LXRs *via* induction of sterol regulatory element-binding protein 1c (SREBP-1c), which is able to bind to the E-box element on the IL-17 promoter and to physically interact with the aryl hydrocarbon receptor (AHR), inhibiting its transcriptional activity (28). Interestingly, the activity of LXRs mediated by oxysterols can also modulate pro-inﬂammatory responses in microglial and astrocytes (26, 27) possibly contributing to ameliorate inflammation.

LXRs have also been hypothesized as a possible therapeutic target for rheumatoid arthritis (RA), a chronic autoimmune disorder characterized by inﬁltration of inﬂammatory leukocytes in the synovial compartment, which causes cartilage and bone damage (87). Initial conflicting reports have described both protective and promoting actions of LXRs-mediated pathways in murine models of inflammatory arthritis. LXR agonists such as T0901317 or GW3965, attenuated the symptoms, decreasing the production of pro-inﬂammatory cytokines in different murine collagen-induced arthritis (CIA) models (23, 31, 32, 88) and suppressed inflammatory gene expressions in RA fibroblast-like synoviocytes (35). By contrast, other reports described increased inflammation and cartilage destruction mediated by ligand activated LXRs (TO901317 or GW3965) in CIA models and found that LXR pathways are significantly upregulated in RA synovial macrophages. Interestingly, in these models the activity of both LXR isoforms was required in control mice to induce the progression of inflammation, in respect to single LXRα^-/-^ or LXRβ^-/-^ mice (33), thus implying overlapping and exclusive effects in these models. Moreover, activation of LXRs by ligands present within synovial fluids enhanced TLR-driven cytokine and chemokine secretion, suggesting a novel mechanism that can promote RA synovitis (33, 34, 89).

In a different scenario, both LXR subtypes are expressed in human and murine colon and were described to mediate anti-inﬂammatory eﬀects in colon epithelial cells (36). Furthermore, in a murine experimental model of intestinal bowel disease (IBD), it was reported that LXR-deﬁcient mice were more susceptible to dextran sodium sulphate (DSS) and 2,4,6-trinitrobenzenesulfonic acid (TNBS)-induced colitis. In this regard, the activation of LXRs can suppress Th1 and Th17 polarization *in vitro*, lowering the expression of their secreted pro-inﬂammatory cytokines and promoting differentiation of protective gut-associated regulatory T cells in mice, where systemic LXR activation was obtained by oral treatment with the LXR agonist GW3965 (37). These data confirmed a dual role of LXR in the control of inflammation by the suppression of pro-inflammatory T cells and the parallel induction of regulatory T cells.

## LXRs as Regulators of Lipid Metabolism, Cancer Progression, and Antitumor Immunity

Genes involved in cholesterol homeostasis are often mutated or dysregulated in cancer cells (10, 90). A higher intracellular cholesterol level due to an enhanced uptake by LDLRs, a decreased efflux by ABC transporters and the upregulation of *de novo* synthesis can sustain the metabolic need for cancer cell proliferation (90–93), and accumulation of cholesterol has been described in many types of tumors (9, 49, 91–93).

Cells usually obtain cholesterol *via* different mechanisms including direct synthesis *via* the transcriptional activity of SREBPs, which promote the transcription of enzymes involved in cholesterol and fatty acid biosynthesis [i.e., 3-hydroxy-3-methylglutaryl-coenzyme A reductase (HMG-CoA) reductase] (94, 95). In this regard, the recent use of HMG-CoA inhibitors (Statins) to block the mevalonate pathway and cholesterol *de novo* biosynthesis showed promising results (96). However, cancer cells often gain selective proliferative advantage by enhancing LDLR-mediated uptake of exogenous cholesterol (38), rendering these therapies often unsuccessful. Perhaps, one of the best characterized examples of cancer cholesterol addiction is glioblastoma multiforme (GBM). The treatment of these cancer cells with LXR agonists induced degradation of LDLR and increased apoptosis in glioblastoma cells expressing mutant epidermal growth factor receptor (EGFR), where tumor growth and survival is strongly dependent on SREBP-1-mediated lipogenesis (38). Moreover, triggering of LXRs increases cellular cholesterol efﬂux by ABCA1, lowering its levels and inducing severe GBM cell death. Accordingly, LXR agonists (e.g., LXR-623) prolonged survival of mice models bearing GBM, indicating that targeting cholesterol metabolism may be a promising strategy in the treatment of this cancer (39, 97).

Pharmacological studies on various types of cancer models such as prostatic carcinoma, colon, mammary and skin cancer have shown that the activation of LXRs generates anti-proliferative effects due to the destruction of growth signalling pathways and to the activation of pro-apoptotic signals (9). LXRs can reduce the expression/activity of cell-cycle regulators, as shown for S-phase Kinase associated protein (SPK2) in cancer cell lines (40) and, at the same time, are able to induce the expression of cell-cycle inhibitors as demonstrated for p21 and p27 (cyclin-dependent kinase inhibitors) in prostate and ovarian cancer cells, with a concomitant reduction in phospho-RB protein levels (41, 98). Moreover, in mouse models, activation of LXRs delayed the progression of androgen-dependent tumors towards androgen independence (41, 42).

In addition to these direct activities on cancer cell metabolism and survival, in the last few years experimental evidences have highlighted the importance of LXRs in anti-tumor immune responses. In this context, the role of LXR activation is still controversial. Several tumors can produce oxysterols that play an essential role in cholesterol homeostasis by activating LXRs (99, 100), and many of these metabolites can have antiproliferative activity in cancer cells (101). However, oxysterols can also inhibit the expression of CCR7 on DCs, a chemokine receptor critical for the migration of DCs to tumor-draining lymph nodes (44). Circulating and tumor-derived oxysterols have been also described to recruit pro-tumor neutrophils and to increase neo-angiogenesis and immunosuppression in a CXCR2-dependent and LXR-independent manner (6, 102). This highlights the capability of selected oxysterols to regulate a broad range of pro-tumor activities—depending on the LXR isoform expressed by the tissue from which tumor cells originate and on the surrounding microenvironment. Moreover, in breast cancer, 27-hydroxycholesterol has been shown to act as an estrogen receptor agonist inducing tumor growth and metastasis (103). On the other hand, LXRs were reported to control cancer cell growth by inducing LXRβ-dependent pyroptosis of cancer cells and the activation of LXRα in macrophages, promoted the phagocytosis of dying cancer cells (45). More recently, in different mouse cancer models treated with specific LXRs agonists (i.e., RGX-104), has been observed a slower tumor growth which correlated with a decreased expansion of myeloid derived suppressor cells (MDSCs); these data were also validated in cancer patients, in a multicentre dose escalation phase 1 trial (43). Moreover, RGX-104 also partially abrogated the immunosuppressive effects of radiotherapy in a murine model of Non-Small-Cell Lung Carcinoma (NSCLC) (104). Mechanistically, the induction of ApoE, a transcriptional target of LXR, can induce MDSC depletion by triggering the low-density lipoprotein receptor-related 8 (LRP8) receptor on these cells, and potentiate activation of cytotoxic lymphocytes. In these settings, activation of LXRs together with PD-1 inhibition, improved the efﬁcacy of cytotoxic T lymphocyte (CTL) and natural killer (NK) cells from cancer patients (43). In a different scenario, LXRs activation could upregulate MHC class I polypeptide-related sequence-A (MICA) and MICB expression in multiple myeloma cells, two ligands of the NK cell-activating receptor NK group 2 member D (NKG2D), by enhancing MICA promoter activity and inhibiting MICB degradation in lysosomes, thus improving NK cell-cytotoxicity (46).

## Conclusion

**Figure 2:**
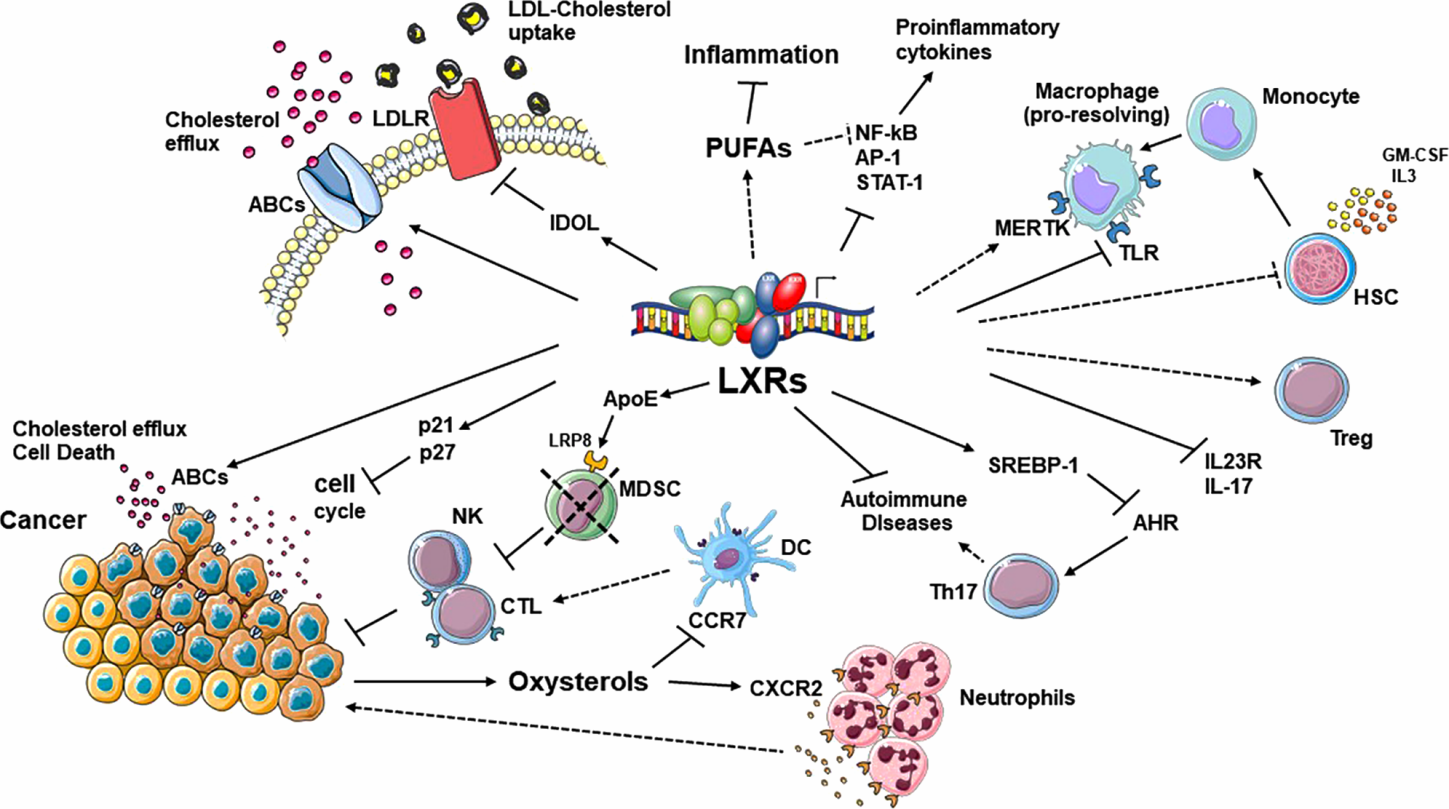
Summary of the most relevant pathways regulated by LXRs in the context of cholesterol homeostasis, inflammation, autoimmunity and tumor progression. These NRs act in a cell-, tissue- and context-dependent manner. In addition, many LXR-dependent and -independent pleiotropic effects mediated by oxysterols, produced in inflamed or tumoral microenvironments, add an additional level of complexity to these regulatory pathways. ABC, ATP binding cassette; AHR, aryl hydrocarbon receptor; APOE, apolipoprotein E; CTL, cytotoxic T lymphocyte; DC, dendritic Cell; DSS, IDOL, inducible degrader of the LDL-receptor; IRF3, interferon regulatory factor 3; LBD, ligand binding domain; PUFA, long-chain polyunsaturated fatty acid; LDLR, low-density lipoprotein receptor; LRP8, low-density lipoprotein receptor-related 8; MDSC, myeloid derived suppressor cells; MERTK, MER proto-oncogene tyrosine kinase; NK, natural killer; SREBP-1, sterol regulatory element-binding protein 1; TLR, Toll-like receptor.

The implication of cholesterol metabolism in the control of inflammatory diseases and cancer progression is the object of an interesting and controversial debate. Our increasing knowledge of the different roles mediated by LXRs in lipid homeostasis supports the idea that lipid metabolism and inflammation are closely connected and that their crosstalk is crucial for the evolution of different inflammatory diseases and, more in general, in the regulation of the immune response. The involvement of specific pathways regulated by LXRs during tumor progression and the possibility to pharmacologically modulate LXR activity, as an additional weapon for cancer therapy and for immunotherapy, have opened new therapeutic possibilities in this context. However, the activities of these NRs are often cell-, tissue-, and context-dependent, which makes it difficult to fully characterize their effects in disease conditions and to optimize specific therapeutic interventions in inflammatory disorders or in cancer therapy. In addition, many LXR-dependent and -independent pleiotropic effects of oxysterols produced in inflamed or tumoral microenvironments have been described in recent years, adding additional levels of complexity to these regulatory pathways (**Figure 2**). Another important issue is whether synthetic ligands that uncouple the anti-inflammatory and anti-cancer effects of LXRs from their role in cholesterol homeostasis can be developed. This is particularly important also in the context of different metabolic disorders with increased risk of developing diseases such as type 2 diabetes or cardiovascular disease, where beneficial effects of LXRs have been described (105). At the moment, different synthetic LXRs agonists have been optimized; however, their clinical application is limited by undesirable hyperlipidemic effects and other adverse side effects encountered in the central nervous system (106–111). The future development of isoform- and/or tissue-speciﬁc LXR modulators and the possibility to target LXR-interacting co-factors involved in LXR transcriptional activation will open new therapeutical possibilities for treating these diseases.

## Author Contributions

MB and SP made substantial contributions to conception and design of the review. MC organized the study, together with AS, contributed to revision, and approved the submitted version. All authors contributed to the article and approved the submitted version.

## Funding

This work was supported by grants from MIUR Ateneo 2018 and PRIN 2017 to MC. The funders had no role in the study design, data collection and analysis, decision to publish, or preparation of the manuscript.

## Conflict of Interest

The authors declare that the research was conducted in the absence of any commercial or financial relationships that could be construed as a potential conflict of interest.
